# Supplementary material for: Phenotype-Driven Diagnostic of PTEN Hamartoma Tumor Syndrome: Macrocephaly, But Neither Height nor Weight Development, Is the Important Trait in Children
Source: Cancers (Basel). 2019 Jul 11;11(7):975. doi: 10.3390/cancers11070975 (PMC6679020; doi:10.3390/cancers11070975)
Supplement: Supplementary file 1 [file cancers-11-00975-s001.pdf]

Supplementary Material

Phenotype-Driven Diagnostic of *PTEN* Hamartoma Tumor Syndrome: Macrocephaly, But Neither Height nor Weight Development, is the Important Trait in Children

Michaela Plamper, Bettina Gohlke, Felix Schreiner and Joachim Woelfle

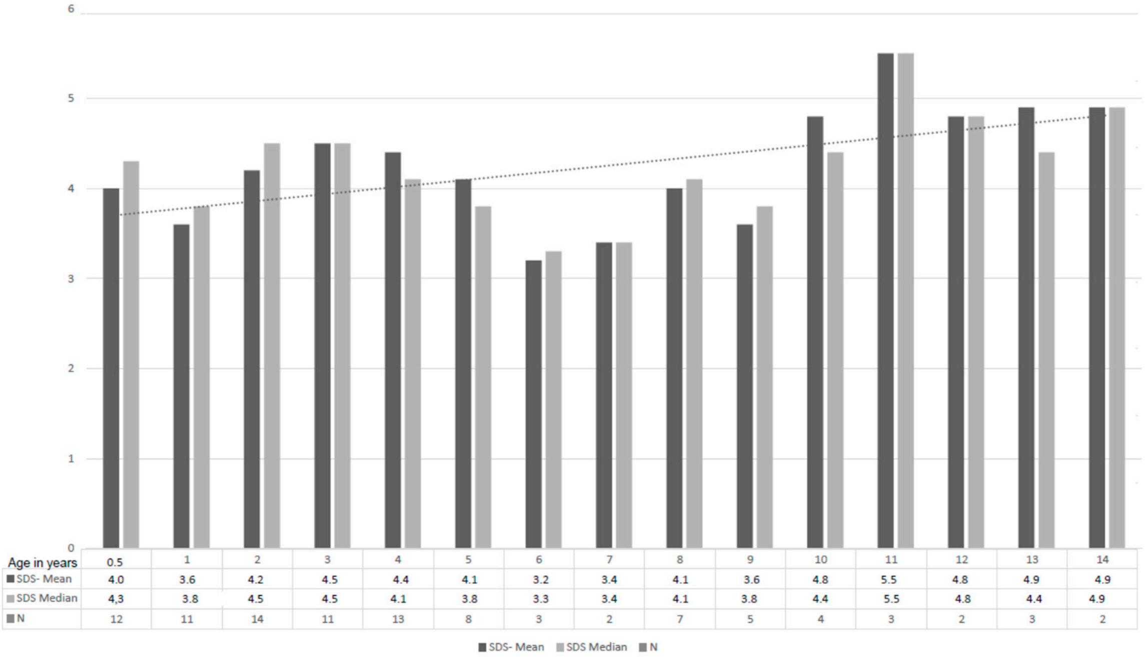

Age in years	0.5	1	2	3	4	5	6	7	8	9	10	11	12	13	14
SDS- Mean	4.0	3.6	4.2	4.5	4.4	4.1	3.2	3.4	4.1	3.6	4.8	5.5	4.8	4.9	4.9
SDS Median	4,3	3.8	4.5	4.5	4.1	3.8	3.3	3.4	4.1	3.8	4.4	5.5	4.8	4.4	4.9
N	12	11	14	11	13	8	3	2	7	5	4	3	2	3	2

Figure S1. Head circumference in male PHTS patients (SDS; Median and Mean).

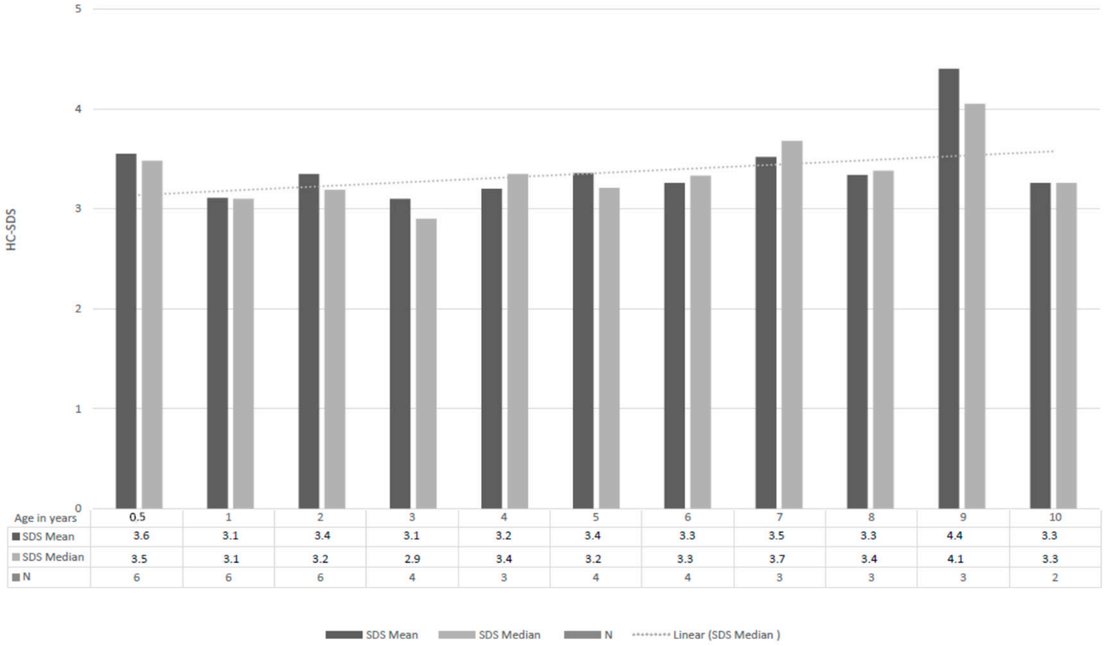

Age in years	0.5	1	2	3	4	5	6	7	8	9	10
SDS Mean	3.6	3.1	3.4	3.1	3.2	3.4	3.3	3.5	3.3	4.4	3.3
SDS Median	3.5	3.1	3.2	2.9	3.4	3.2	3.3	3.7	3.4	4.1	3.3
N	6	6	6	4	3	4	4	3	3	3	2

Figure S2. Head circumference in female PHTS patients (SDS, Median and Mean).

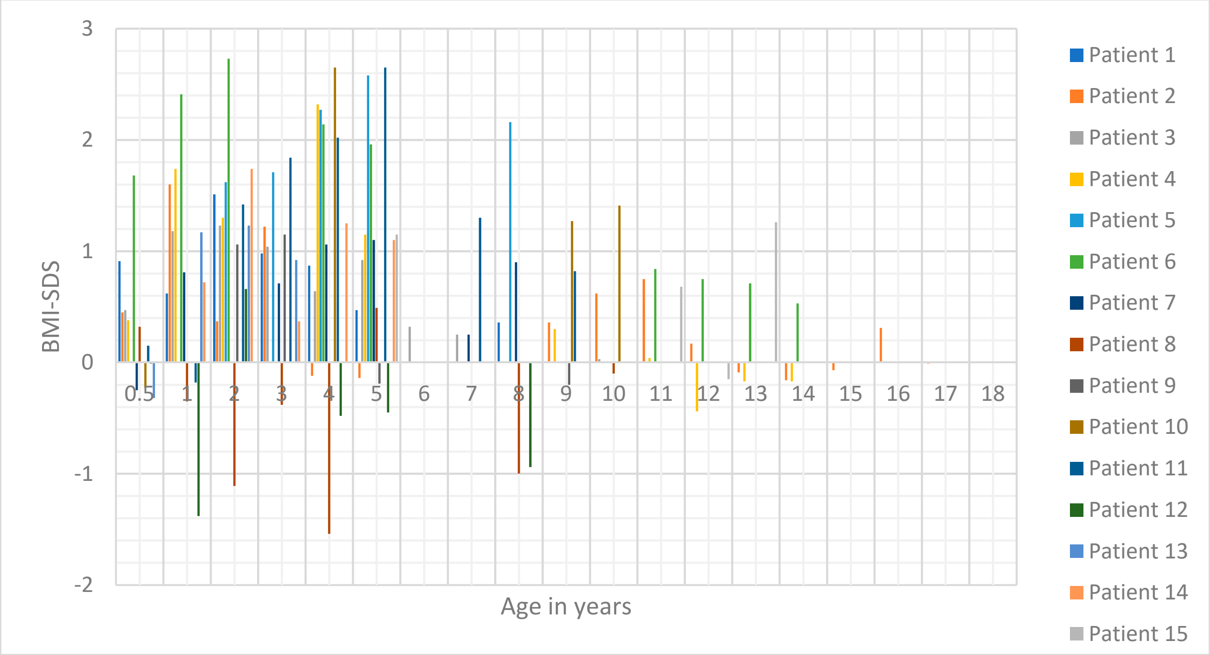

Figure S3. BMI-SDS of male PHTS patients in comparison to German Reference growth charts [11].

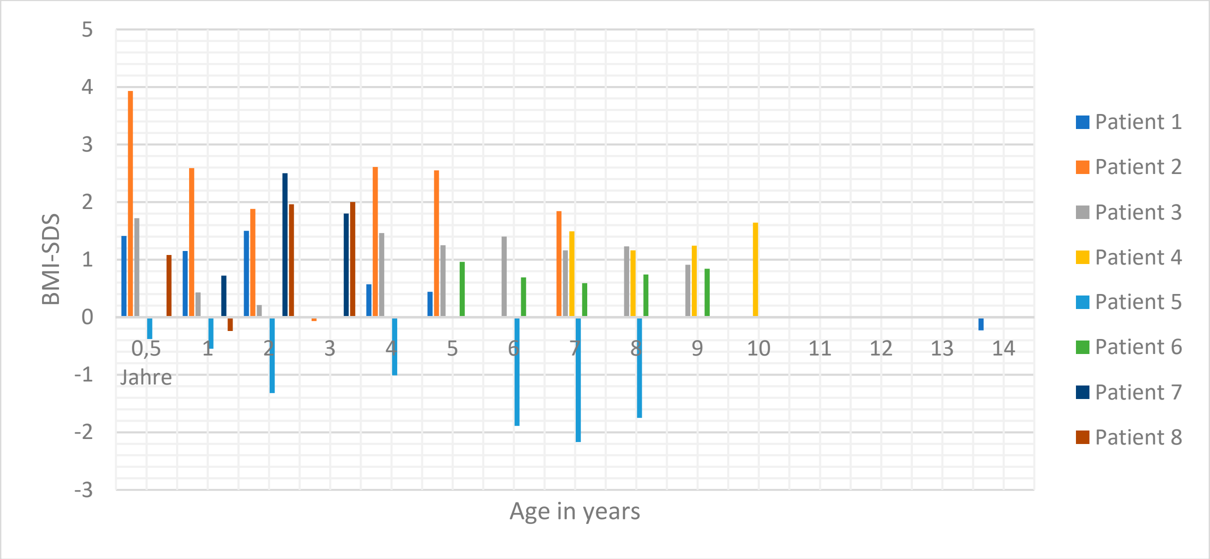

Figure S4. BMI-SDS of female PHTS patients in comparison to German Reference growth charts [11].

© 2019 by the authors. Licensee MDPI, Basel, Switzerland. This article is an open access article distributed under the terms and conditions of the Creative Commons Attribution (CC BY) license (http://creativecommons.org/licenses/by/4.0/).
